# Laser Interstitial Thermal Therapy as a Treatment Option for Malignant Peripheral Nerve Sheath Tumor Metastases to the Brain: A Case Report

**DOI:** 10.7759/cureus.53855

**Published:** 2024-02-08

**Authors:** Annie Pico, Isabel L Bauer, Kristin Nosova, Ashley Kern, Robert Bina

**Affiliations:** 1 Neurological Surgery, University of Arizona College of Medicine - Phoenix, Phoenix, USA; 2 Neurosurgery, University of Arizona College of Medicine - Phoenix, Phoenix, USA; 3 Neurosurgery, Banner University Medical Center - Phoenix, Phoenix, USA

**Keywords:** peripheral nerve disorders, malignant peripheral nerve sheath tumors, stereotactic laser ablation, laser interstitial thermal therapy, malignant peripheral nerve sheath tumor

## Abstract

We present the unique case of a 60-year-old female with neurofibromatosis type 1 (NF1) who underwent laser interstitial thermal therapy (LITT) for metastatic malignant peripheral nerve sheath tumor (MPNST) of the brain. She presented to the emergency room complaining of one week of dysarthria and facial droop. An MRI of the brain demonstrated a homogeneously enhancing left frontal mass; although rare, given her history of pulmonary MPNST, brain invasion was considered likely. No generally accepted guidelines for the treatment of MPNST with cerebral metastases exist; however, LITT was chosen due to tumor morphology and proximity to eloquent brain structures. She did not experience any new or worsening neurological deficits post-operatively. Post-ablation MRI showed white matter edema surrounding the lesion, which is consistent with previously reported cases. This case illustrates the use of LITT for cytoreduction for rare brain metastases located near eloquent brain structures.

## Introduction

Malignant peripheral nerve sheath tumors (MPNST) are soft tissue neoplasms arising from peripheral nerves that arise spontaneously or with neurofibromatosis type 1 (NF1). They occur most frequently in the proximal extremities and have a tendency to recur and metastasize. The most common sites of metastasis are the lungs, bones, lymph nodes, and liver. Although metastasis is common for MPNST, very few intracranial metastases have been reported. As of 2021, 29 cases of intracranial MPNST metastasis have been reported in the literature, eight of which occurred in patients with NF1 [1,2]. All of these patients were treated with surgical resection, whole-brain radiation, chemotherapy, and/or radiotherapy, with an average reported survival time of 5.9 months after diagnosis. We report the first use of laser interstitial thermal therapy (LITT) for the treatment of metastatic MPNST. 

## Case presentation

Case

**Figure 1:**
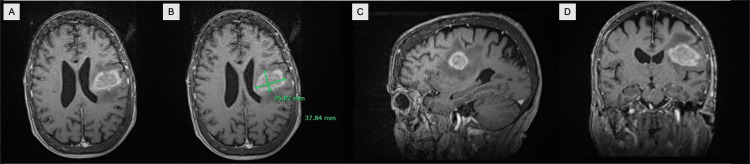
Preoperative MRI. A: T1-weighted MRI with contrast in the axial plane showing an enhancing mass in the inferior left frontal lobe with vasogenic edema. B: T1-weighted MRI with contrast in the axial plane showing an enhancing mass measuring 25.02mm x 37.84mm in the inferior left frontal lobe with vasogenic edema. C: T1-weighted MRI with contrast in the sagittal plane showing an enhancing mass in the inferior left frontal lobe with vasogenic edema. D: T1-weighted MRI with contrast in the coronal plane showing an enhancing mass in the inferior left frontal lobe with vasogenic edema.

**Figure 2:**
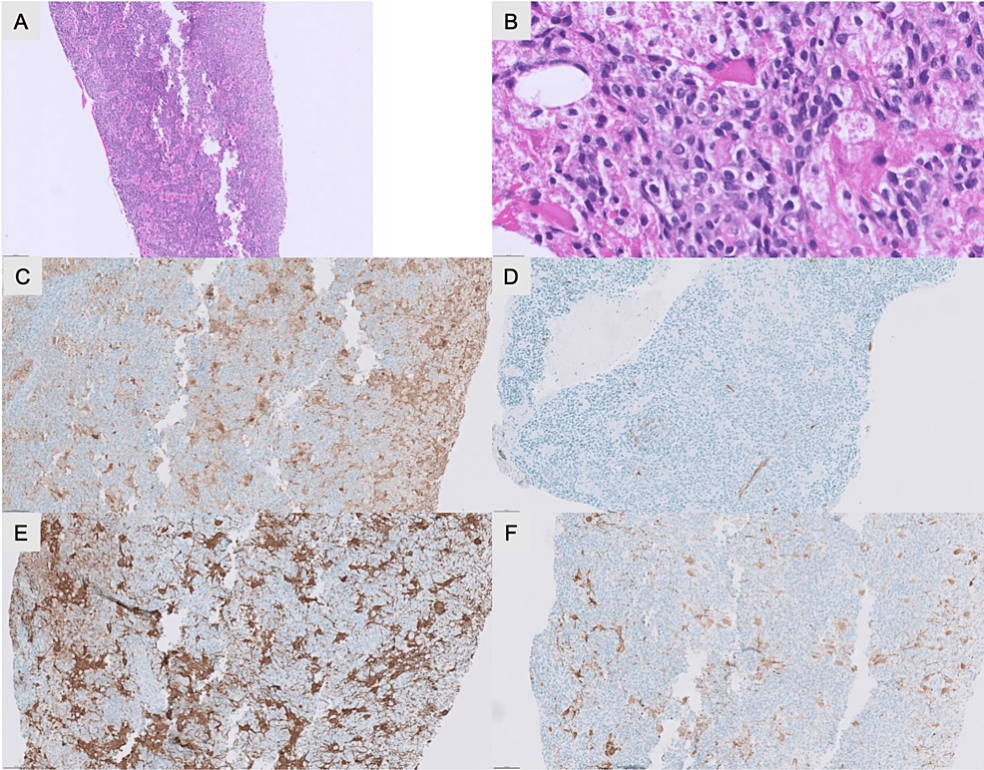
Histology. A: Hematoxylin & eosin (H&E) stain at low power showing high-grade malignancy with small blue cell and spindle cell features. B: H&E stain at high power showing high-grade malignancy with small blue cell and spindle cell features. C: S100 stain negative. D: CD34 stain negative. E: Glial fibrillary acidic protein (GFAP) stain negative. F: Desmin stain showing neuronal/rhabdoid cells.

This patient’s medical history is significant for NF1 and prior radiation and surgical resection of the primary-a left lower extremity MPNST-in August 2021. The tumor recurred locally and metastasized to the lungs in 2022 and was treated with adriamycin, ifosfamide, and mesna (AIM). On January 22, 2023, she presented to the emergency room complaining of one week of dysarthria and facial droop. Physical examination revealed multiple neurofibromas, dysarthria, right central facial droop, and stable left lower extremity weakness. An MRI of the brain demonstrated a homogeneously enhancing left frontal mass measuring 25.02 mm x 37.84 mm (Figure 1). A biopsy was performed prior to the LITT for tissue diagnosis; the histopathological results of this biopsy are shown in Figure 2, confirming the diagnosis of metastatic MPNST. Laser interstitial thermal therapy (LITT) was chosen for cytoreduction due to the tumor’s size and proximity to eloquent brain structures. Improved peritumoral blood-brain barrier (BBB) permeability after LITT was also a consideration, in case adjuvant chemotherapy was pursued as part of the treatment regimen [3].

Surgery

On February 10, 2023, intraoperative stereotactic computed tomography head (CTH) was obtained and merged with a preoperative MRI with a pre-planned trajectory, and stereotactic coordinates were generated. A 6.5-mm left temporal burr hole was made through the Varioguide arm, and a Monteris VUE ceramic bolt was placed. The patient was transported under general anesthesia to MRI and Monteris NeuroBlate SideFire (Monteris Medical, Minneapolis, MN) 3.3 mm directional laser fiber was introduced into the stereotactic probe. On the long axis of the growing tumor, four isocenters of laser ablation were done up to the white matter tracts at the back of the growth. The tumor was subtotally ablated, preoperative volume was 12.89 cm^2^ and post-operative volume ablated was 12.34 cm^2^, which is 95% ablation. The probe and laser were removed, and the skin was closed with staples.

Post-operative course

The tumor was confirmed histologically as MPNST (Figure 2). The patient was discharged post-operatively to skilled nursing facility (SNF) care. Due to insurance barriers and the consequent inability of the SNF to transport her to oncology appointments, she was lost to follow-up until she presented to the ED in status epilepticus with respiratory failure on 3/31. Her family withdrew supportive care on 4/6, and she expired on 4/9, 58 days after LITT.

## Discussion

Laser interstitial thermal therapy, also known as stereotactic laser ablation, is a minimally invasive, cytoreductive technique that can be applied intracranially with the use of intraoperative MRI. This technique works by converting light energy from a laser into thermal energy. The photons from the laser are absorbed by chromophores, causing the chromophores to become excited and release thermal energy, which ultimately results in protein denaturation, cellular necrosis, and tissue coagulation. This approach is useful in the treatment of tumors that cannot be grossly resected for reasons including proximity to eloquent brain structures or deep locations within the parenchyma.

One of the benefits of LITT is the minimally invasive aspect of the approach, which alleviates time delays in commencing adjuvant radiation and/or chemotherapy by addressing concerns about wound healing. Salvage chemotherapy can achieve better efficacy following maximal safe cytoreduction, especially for tumors for which other treatment options have been exhausted [4]. Additionally, several studies have demonstrated that disruption of the local blood-brain barrier (BBB) after LITT has resulted in improved peritumoral concentrations of chemotherapy agents with poor CNS penetration [3].

**Figure 3:**
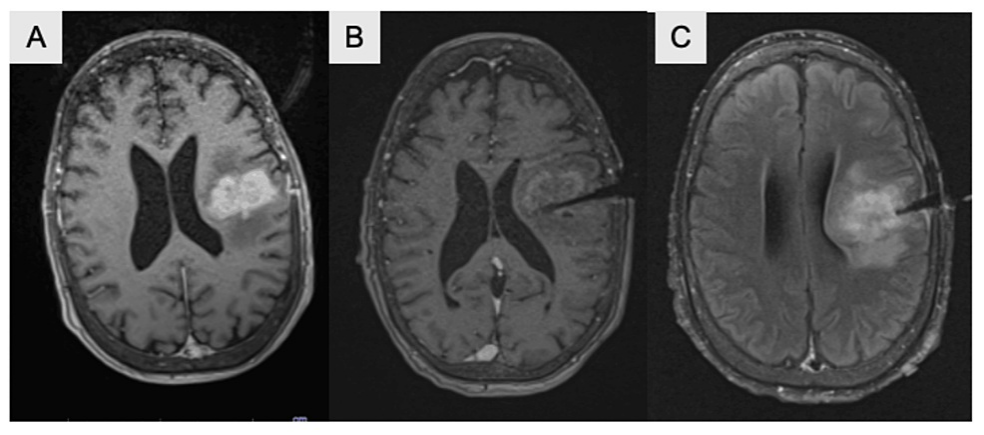
Post-ablation of MRI. T1-weighted MRI with contrast in the axial plane before (A) and after (B) ablation showing ablated region with reduced enhancement compared to preoperative films. (C) T2-weighted FLAIR MRI in the axial plane post-LITT showing an ablated region with increased signal. LITT: laser interstitial thermal therapy; FLAIR: fluid-attenuated inversion recovery.

Complications of LITT include seizures, neurological defects, catheter misplacement, infection, hydrocephalus, hemorrhage, thermal injury, and malignant edema [5,6]. Overall, LITT is generally well-tolerated but can cause malignant edema, especially when used for tumors with a larger volume. Some papers report using LITT in conjunction with resection for larger tumors in an attempt to reduce the possibility of malignant edema, while others recommend avoiding LITT altogether in the treatment of large tumors. In the case of this patient, her post-ablation MRI showed white matter edema surrounding the lesion, as seen in Figure 3, which is consistent with reported postoperative imaging changes. There was no marked change in her neurological deficits post-operatively.

Established indications for the use of LITT include primary and metastatic brain tumors, radiation necrosis, and epilepsy foci [5]. In regards to low-grade gliomas, LITT is used most frequently for unresectable tumors located in or near eloquent brain structures or for patients who cannot tolerate open surgery. For high-grade gliomas, LITT is primarily reserved for treatment-refractory residual or recurrent glioblastoma multiforme (GBM). In most reported cases of the use of LITT for tumor treatment, the tumors are relatively small (volumes less than 10 cm cubed) [5].

LITT plays a unique role in the diagnosis and treatment of enlarging or enhancing lesions following radiation therapy. Since LITT is often performed in conjunction with stereotactic biopsy, this allows for a determination of tumor recurrence versus radiation necrosis with subsequent cytoreductive treatment as needed [5]. Of particular interest in the case of this patient is the use of LITT as a treatment modality for MPNST metastatic to the brain. 

Alternative options to LITT included surgical resection, radiosurgery, and chemoradiation. Griffin et al. found that patients with metastatic MPNST to the brain treated with surgical resection followed by radiation and/or chemotherapy had the longest survival time (12.3 months), followed by whole-brain radiation alone (8.7 months) and surgical resection alone (5.7 months) [2]. Two of the patients in their cohort (n=26) underwent gamma-knife radiosurgery, and one underwent linear accelerator-based stereotactic radiosurgery. These findings suggest that surgical resection followed by adjuvant therapy increased survival time more than any single intervention alone.

Rubino et al. analyzed 26 patients with metastatic MPNST to the brain; twelve underwent surgical resection with or without adjuvant therapy, and seven underwent adjuvant therapy only. Of these, four underwent chemotherapy alone, including two patients with anthracycline-based chemotherapy [1]. In the presented case, the lack of long-term follow-up did not allow for the evaluation of patient survival after LITT. 

Anthracycline-based chemotherapy is the first-line treatment of advanced and metastatic MPNST. Kroep et al. suggested a slight superiority of combination doxorubicin/ifosfamide compared to doxorubicin alone (26.9 weeks versus 17 weeks progression-free survival) [7]. However, neither of these agents can cross the BBB effectively to treat intracranial lesions. Using LITT for cytoreduction prior to administering these agents might disrupt the BBB to improve the efficacy of these agents.

Given the eloquent location and the tumor size, we chose LITT for cytoreduction to avoid significant postoperative neurological deficits, which would have greatly impacted this patient’s performance status, with the possible additional benefit of local BBB disruption, should chemotherapy have been pursued. Awake craniotomy was not considered due to the patient's constitution. 

## Conclusions

We present the first reported use of LITT in the treatment of NF1-associated metastatic MPNST to the brain. Using this minimally invasive approach, we were able to preserve the patient’s neurological function and did not worsen her existing neurological defects. LITT can be considered as a safe and effective option for patients with lesions in eloquent locations or for patients who otherwise are not good surgical candidates. However, further research is needed to clarify and expand specific indications for LITT.
